# Supplementary material for: Structural evolution of nitrogenase states under alkaline turnover
Source: Nat Commun. 2024 Dec 2;15:10472. doi: 10.1038/s41467-024-54713-0 (PMC11612016; doi:10.1038/s41467-024-54713-0)
Supplement: Supplementary file 1 — Supplementary Information [file 41467_2024_54713_MOESM1_ESM.pdf]

Supplementary Information:

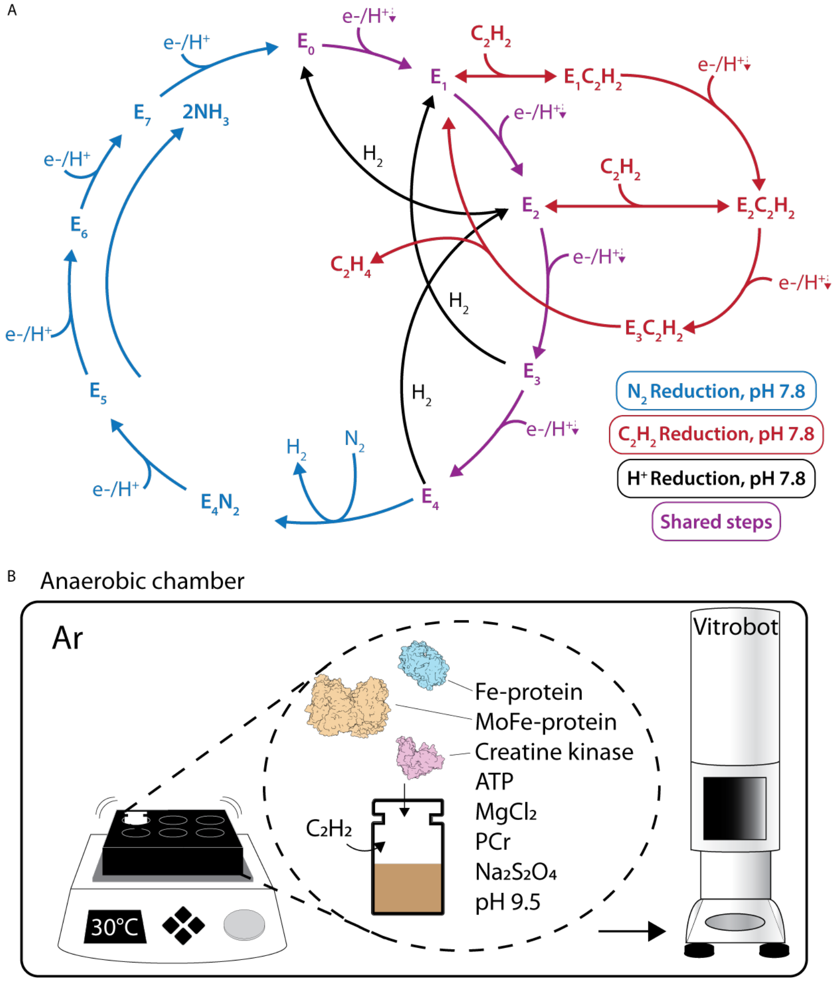

Supplementary Figure 1. Overview of the alkaline acetylene turnover reactions and grid preparation. (A) Kinetic model for the nitrogenase catalyzed reductions of dinitrogen to ammonia (blue), acetylene to ethylene (red), and proton to dihydrogen (black) adapted from Lowe and Thorneley 1990[9,26]. Shared steps are colored in purple. Electron/proton reducing equivalents are represented by e^-/H^+. In the presence of saturating concentrations of C_2H_2, the formation of $E_1C_2H_2$ prevents the formation of the E_2, E_3, and E_4 states, thereby blocking both proton and dinitrogen reductions. The rates of transition from E_n to E_{n+1} will decrease with increasing pH due to the decrease in proton concentration (impacting both the protonation states of active site, and the availability of protons as a substrate), thereby reducing the overall rate of product formation. The use of acetylene reduction at alkaline pH (pH 9.5) restricts the available intermediates from E_0 to E_3, while slowing down the overall reaction rate to extend the pre-steady state period.(B) Schematic of the pipeline for grid preparation of the $MoFe^{Alkaline\text{-}20sec}$, $MoFe^{Alkaline\text{-}5min}$, $MoFe^{Alkaline\text{-}20min}$, and $MoFe^{Alkaline\text{-}60min}$ time points. Black outline indicates that all procedures were carried out within an anaerobic chamber. Surface representations of the MoFe-protein, Fe-protein, and creatine kinase are derived from PDB codes 3U7Q, 2NIP, and 2CRK, respectively. Abbreviations: ATP (adenosine triphosphate), $MgCl_2$ (magnesium chloride), PCr (creatine phosphate), $Na_2S_2O_4$ (sodium dithionite).

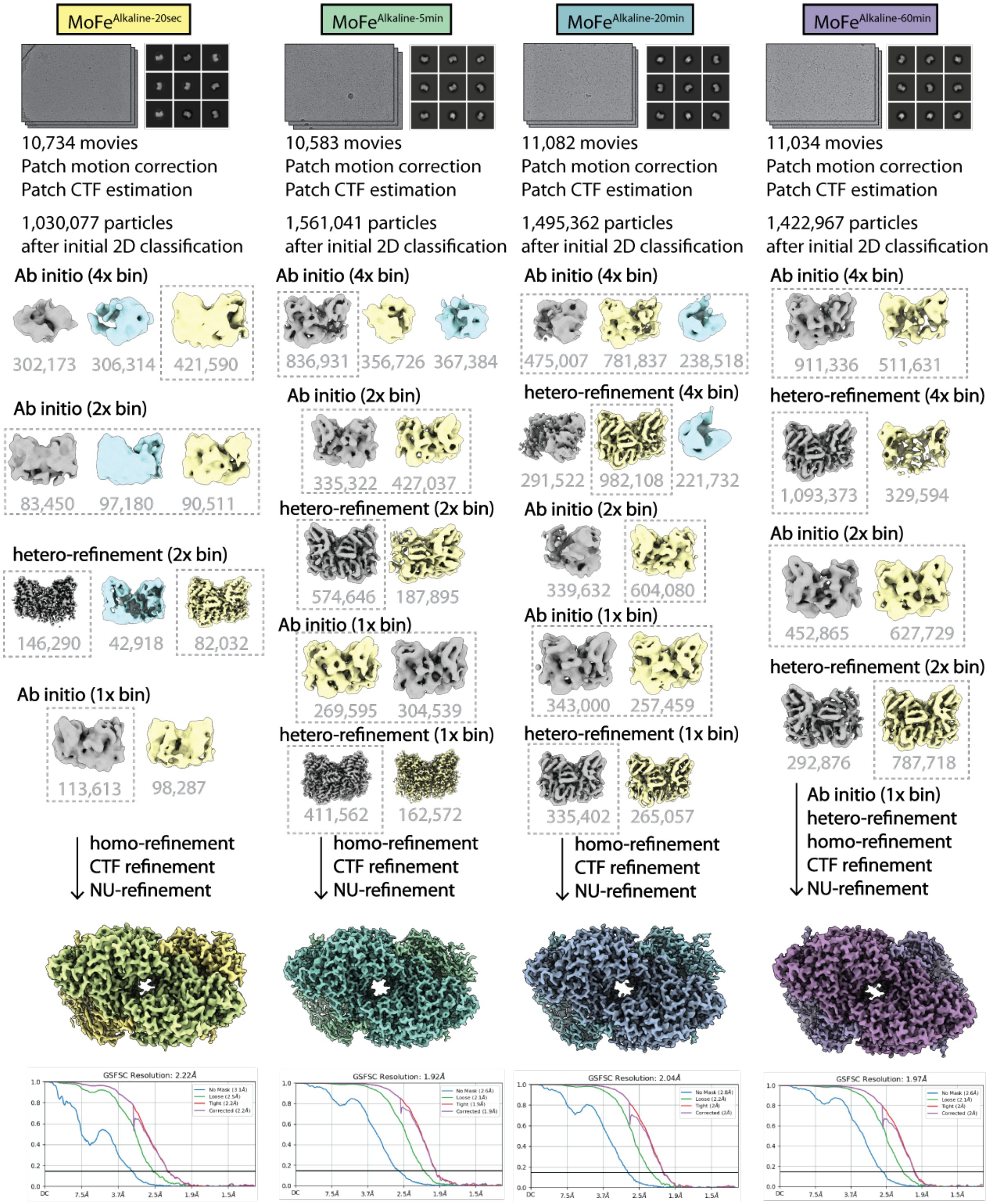

Supplementary Figure 2. CryoEM data processing pipelines for the MoFe$^{Alkaline\text{-}20sec}$, MoFe$^{Alkaline\text{-}5min}$, MoFe$^{Alkaline\text{-}20min}$, and MoFe$^{Alkaline\text{-}60min}$ time points. Processing was completed in cryoSPARC v4.4.1. Workflows are presented from top to bottom.

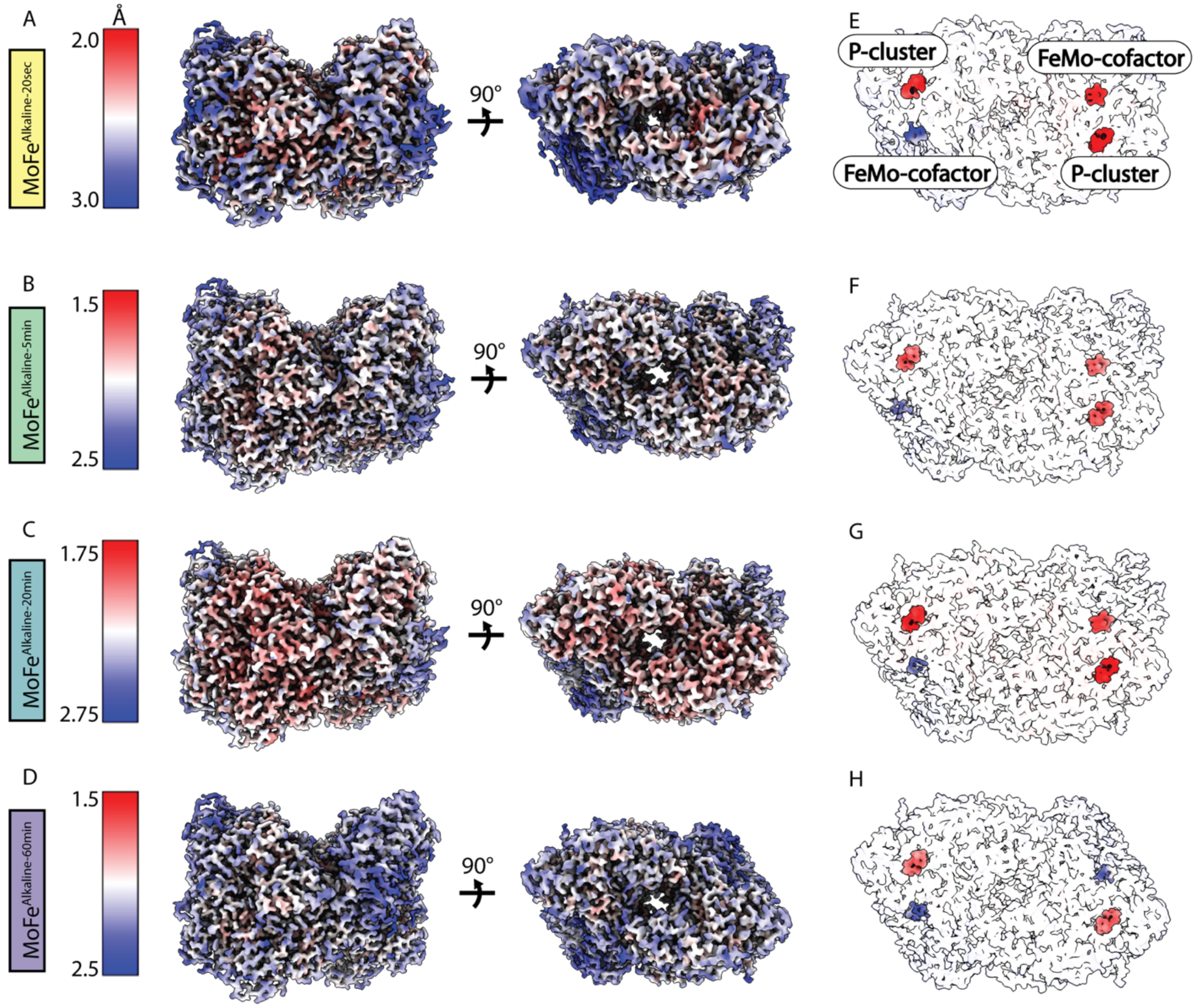

Supplementary Figure 3. Local resolution maps for MoFe$^{Alkaline\text{-}20sec}$, MoFe$^{Alkaline\text{-}5min}$, MoFe$^{Alkaline\text{-}20min}$, and MoFe$^{Alkaline\text{-}60min}$. (A-D) Local resolution estimates performed in cryoSPARC are shown in a red-blue-white palette according to the color key shown to the left of each map in units of Ångstroms. (E-H) Local resolution around the P-cluster and FeMo-cofactor metalloclusters. While the local resolutions of the P-clusters remain relatively constant in all structures, that of the FeMo-cofactor degrades over time.

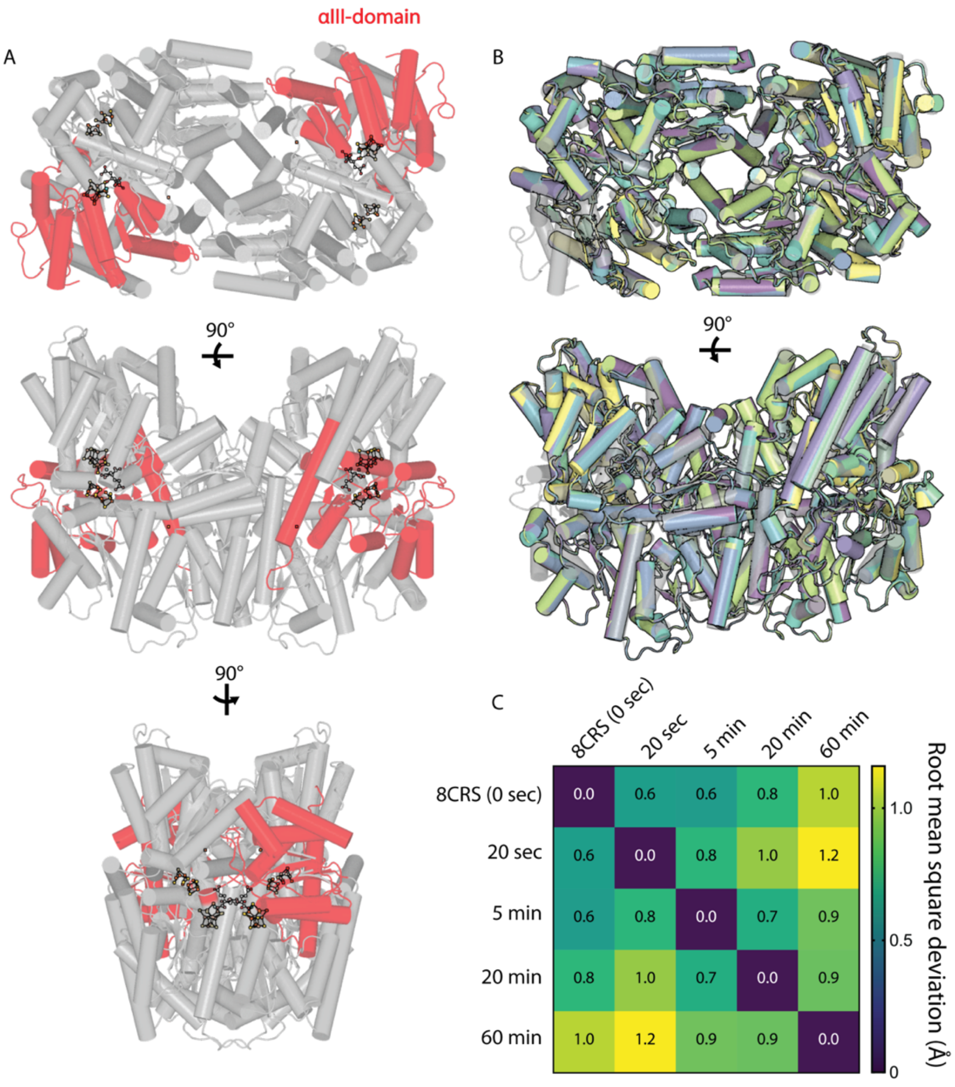

Supplementary Figure 4. Location of the nitrogenase MoFe-protein αIII domain and deviations of the alkaline turnover structures from resting state. (A) Structure of alkaline resting state MoFe-protein (PDB code 8CRS) in gray with the αIII domain highlighted in red. (B) Overlay of the MoFe$^{Alkaline\text{-}20sec}$ (yellow), MoFe$^{Alkaline\text{-}5min}$ (green), MoFe$^{Alkaline\text{-}20min}$ (blue), and MoFe$^{Alkaline\text{-}60min}$ (purple) structures with the alkaline resting state (gray). (C) All atom RMSD in Ångstroms of each time point structure compared with the alkaline resting state structure are displayed as a heat map. Source data are provided as a Source Data file.

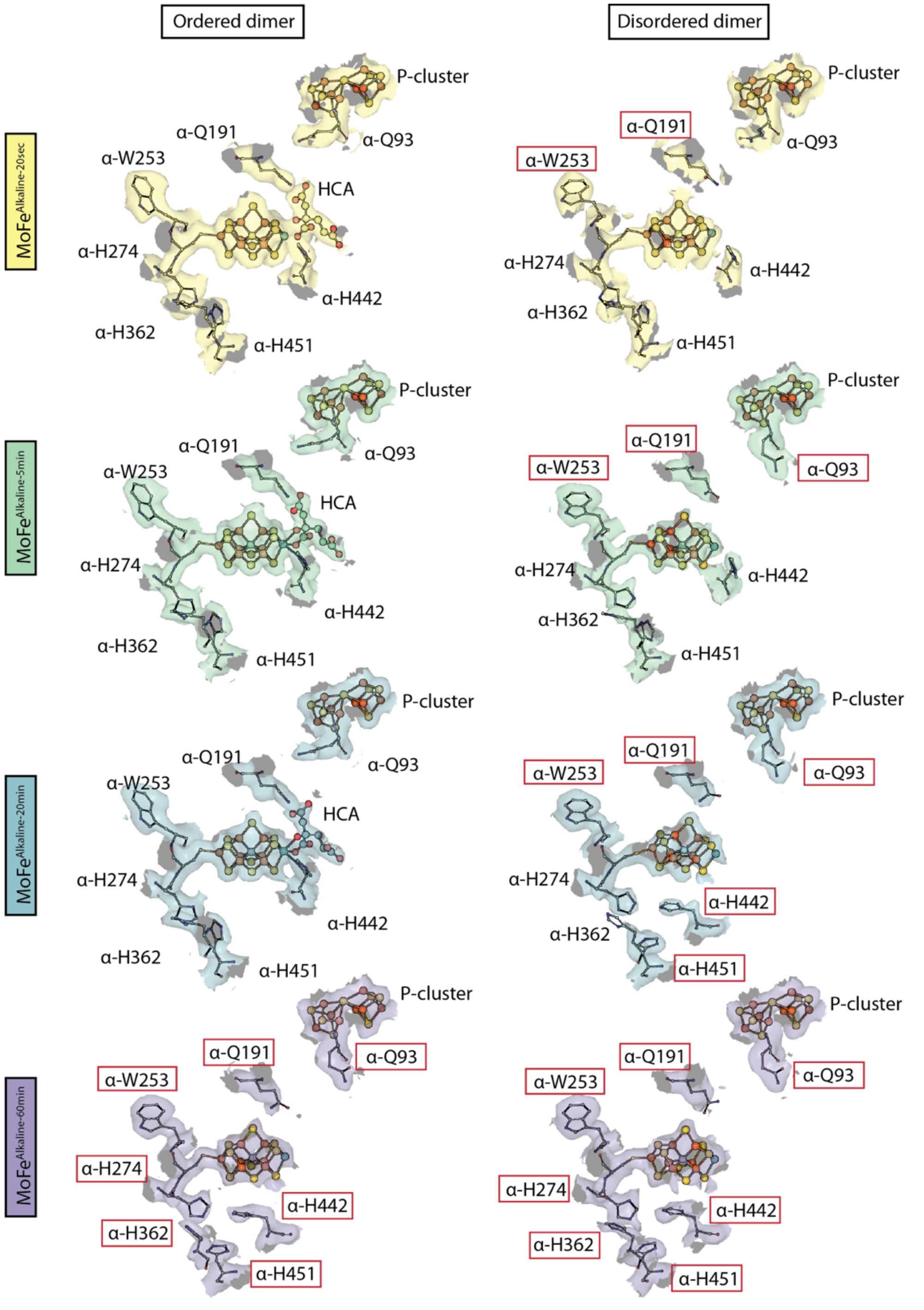

Supplementary Figure 5. CryoEM density for residues adjacent to MoFe-protein metalloclusters. Left panel: CryoEM density for residues in the more ordered active sites of MoFe$^{Alkaline\text{-}20sec}$, MoFe$^{Alkaline\text{-}5min}$, MoFe$^{Alkaline\text{-}20min}$, and MoFe$^{Alkaline\text{-}60min}$, with changes in specific residues in the MoFe$^{Alkaline\text{-}60min}$ time point relative to the resting state structure highlighted by red boxes. Right panel: CryoEM density for residues in the more disordered active sites of MoFe$^{Alkaline\text{-}20sec}$, MoFe$^{Alkaline\text{-}5min}$, MoFe$^{Alkaline\text{-}20min}$, and MoFe$^{Alkaline\text{-}60min}$, with changes in specific residues in all time points highlighted by red boxes.

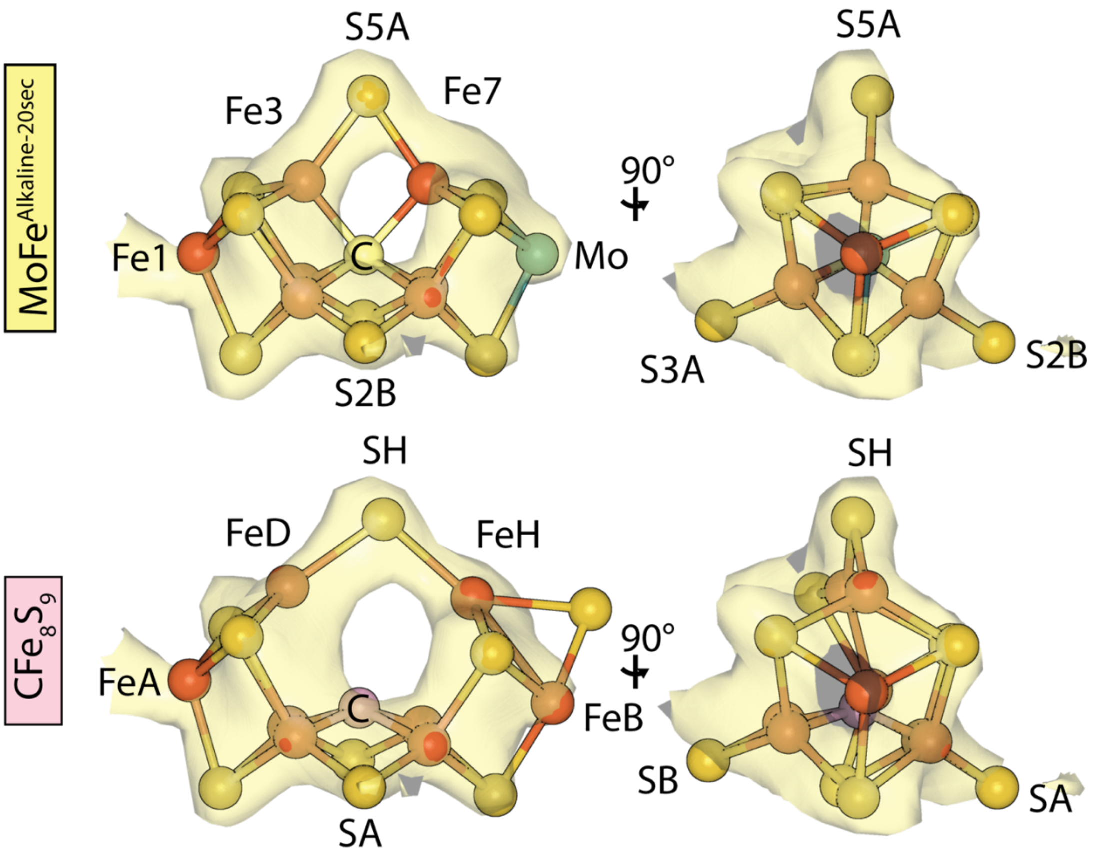

Supplementary Figure 6. CryoEM density of the disordered $MoFe^{Alkaline\text{-}20sec}$ FeMo-cofactor compared with a CFe_8S_9 model. Top panel: Modeled FeMo-cofactor in the $MoFe^{Alkaline\text{-}20sec}$ cryoEM density. Bottom panel: CFe_8S_9 model from McKee[29], in the $MoFe^{Alkaline\text{-}20sec}$ cryoEM density, with atoms labeled as in the original model.

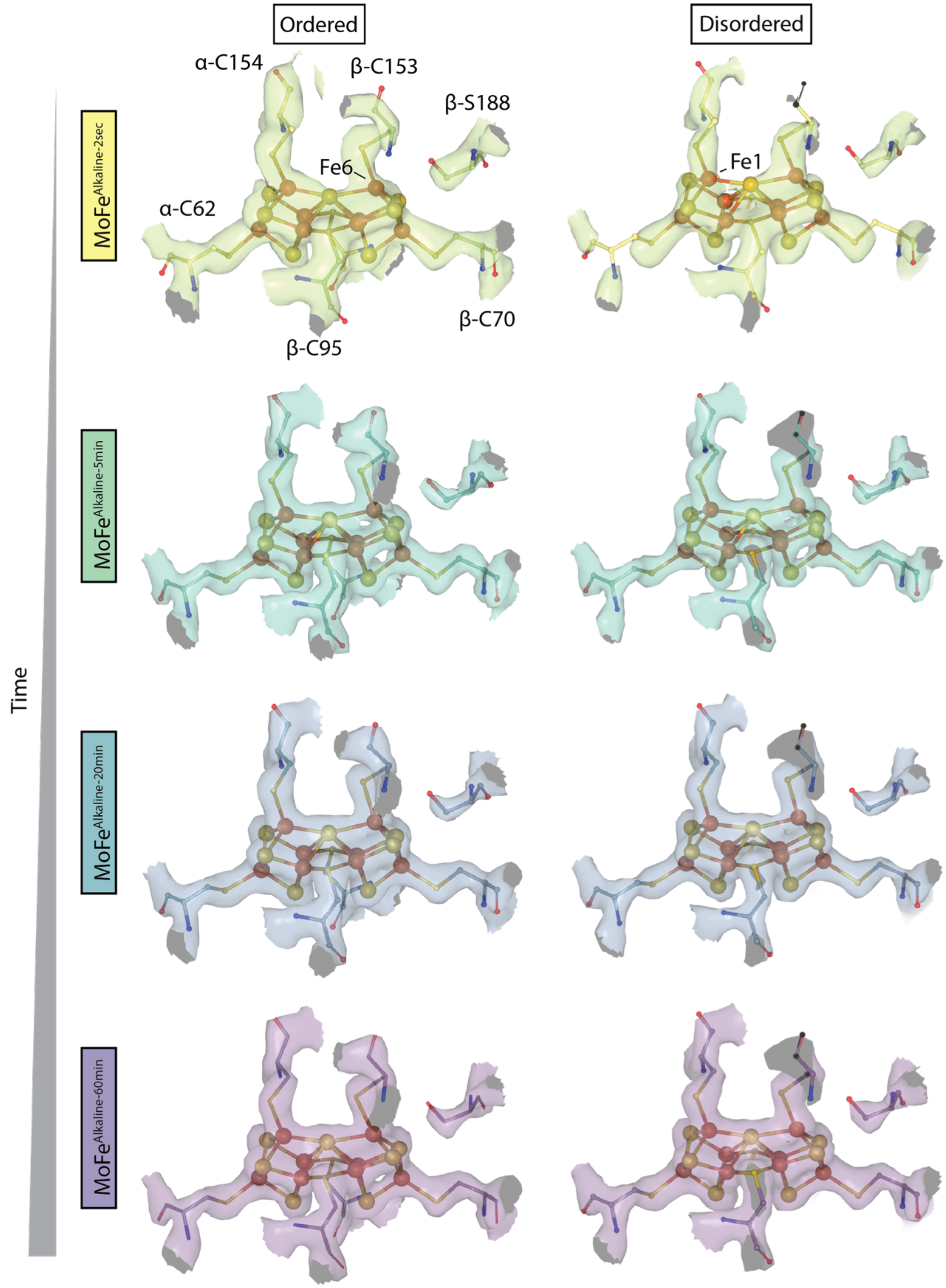

Supplementary Figure 7. CryoEM density for P-clusters within MoFe$^{Alkaline\text{-}20sec}$, MoFe$^{Alkaline\text{-}5min}$, MoFe$^{Alkaline\text{-}20min}$, and MoFe$^{Alkaline\text{-}60min}$. CryoEM density is shown for P-clusters in both ordered and disordered αβ-dimers as well as liganding cysteines and the adjacent serine (β-S188) residue which does not demonstrate any bridging density to the P-clusters.

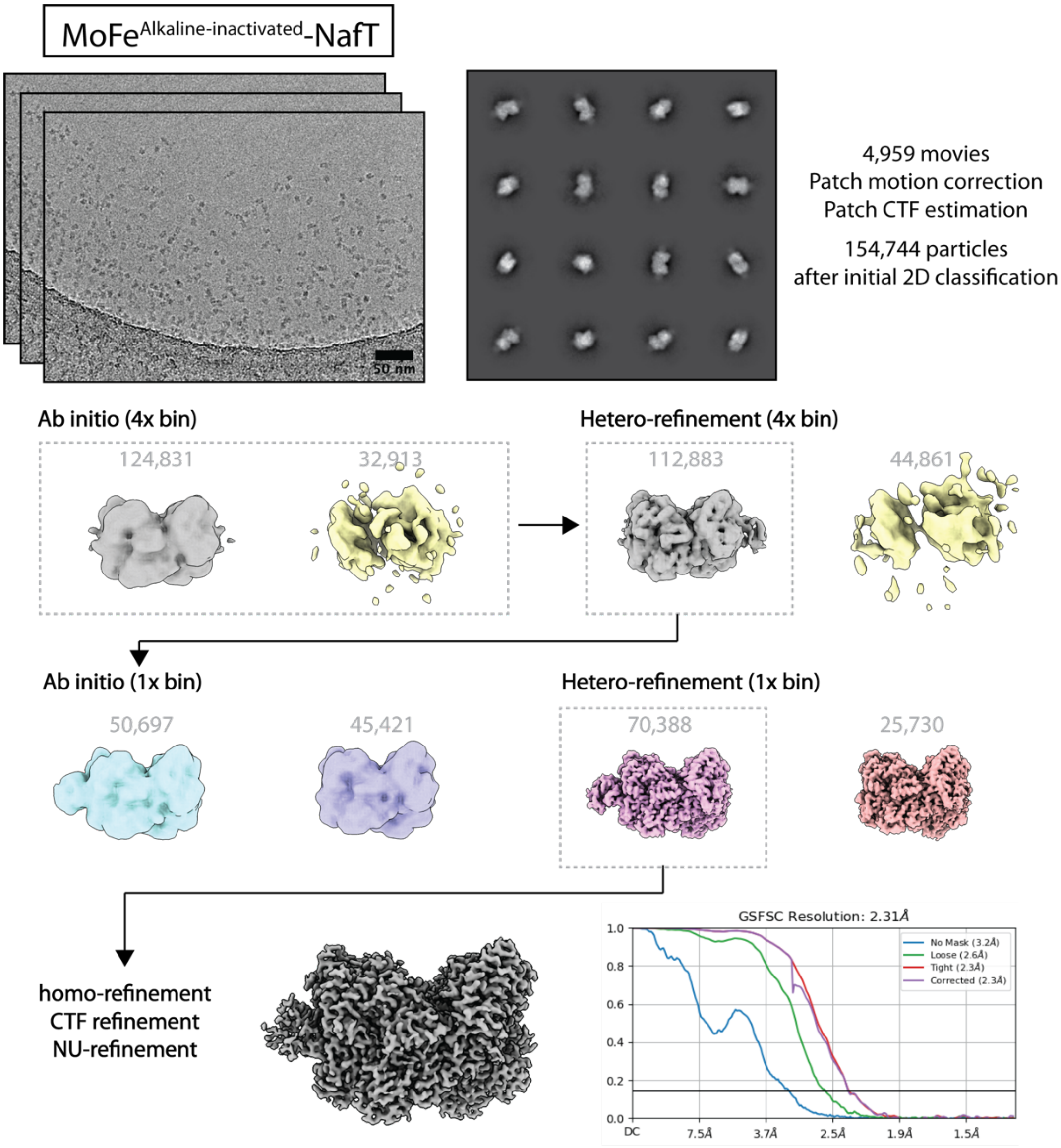

Supplementary Figure 8. CryoEM data processing pipelines for the MoFe$^{Alkaline\text{-}inactivated}$-NafT complex. Processing was completed in cryoSPARC v4.4.1. Numbers shown in gray represent particles.

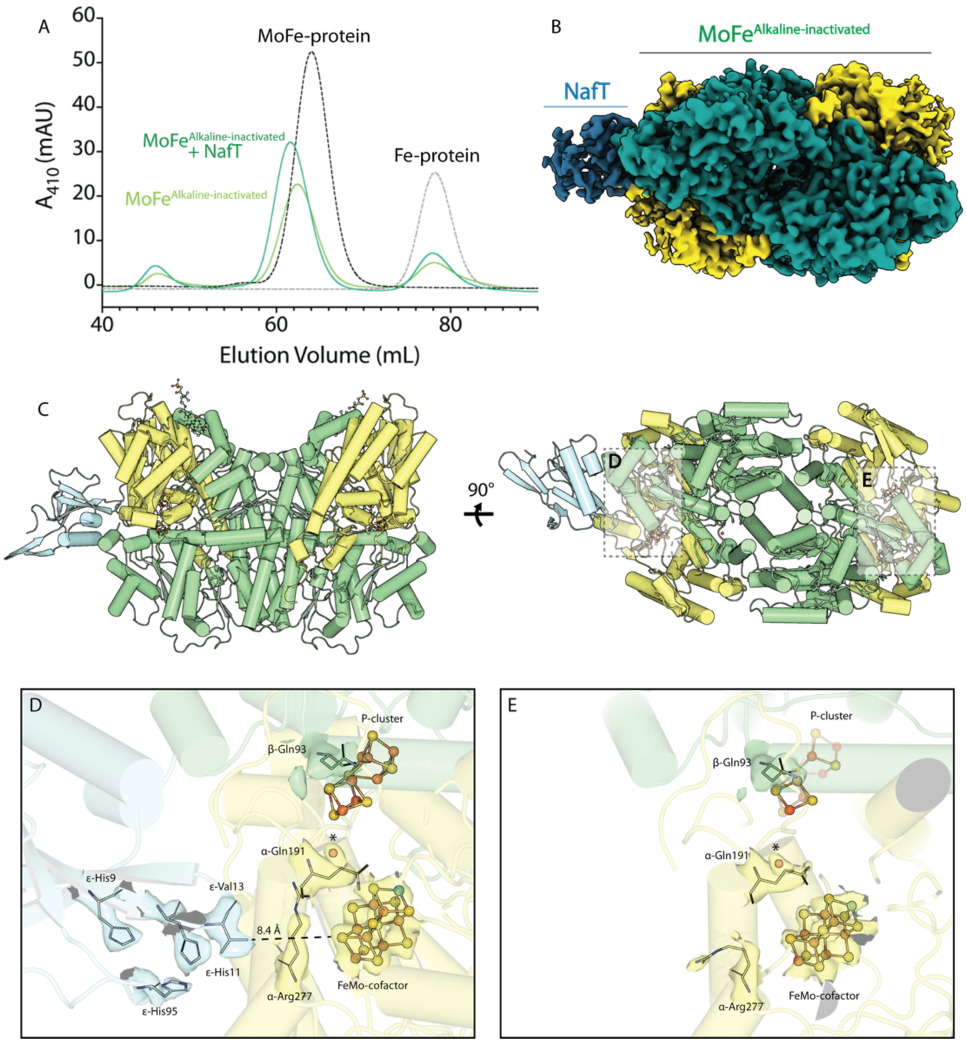

Supplementary Figure 9. CryoEM structure of the MoFe$^{Alkaline-inactivated}$ state in complex with nitrogenase associated factor T (NafT). (A) S.E.C. of MoFe-protein alone (black dashed line), Fe-protein alone (gray dashed line), MoFe$^{Alkaline-inactivated}$ reaction mixture (light green), MoFe$^{Alkaline-inactivated}$ reaction mixture with NafT (dark green). The X-axis represents elution volume in mL and the Y-axis represents absorbance at 410 nm in milli-absorbance units (mAU). Source data are provided as a Source Data file. (B) CryoEM density map for the MoFe$^{Alkaline-inactivated}$-NafT complex at 2.33 Å resolution. (C) Structural model for the MoFe$^{Alkaline-inactivated}$-NafT complex. (D) CryoEM density for the FeMo-cofactor within the αβ-dimer bound to NafT as well as the closest NafT residues ε-Val13, ε-His9, ε-His11, and ε-His95. (E) CryoEM density for the FeMo-cofactor within the opposing αβ-dimer with no bound NafT. The asterisk (*) indicates density for a modeled water which sits in the same position as a sulfur identified in the VFe nitrogenase turnover structure (PDB 6FEA).

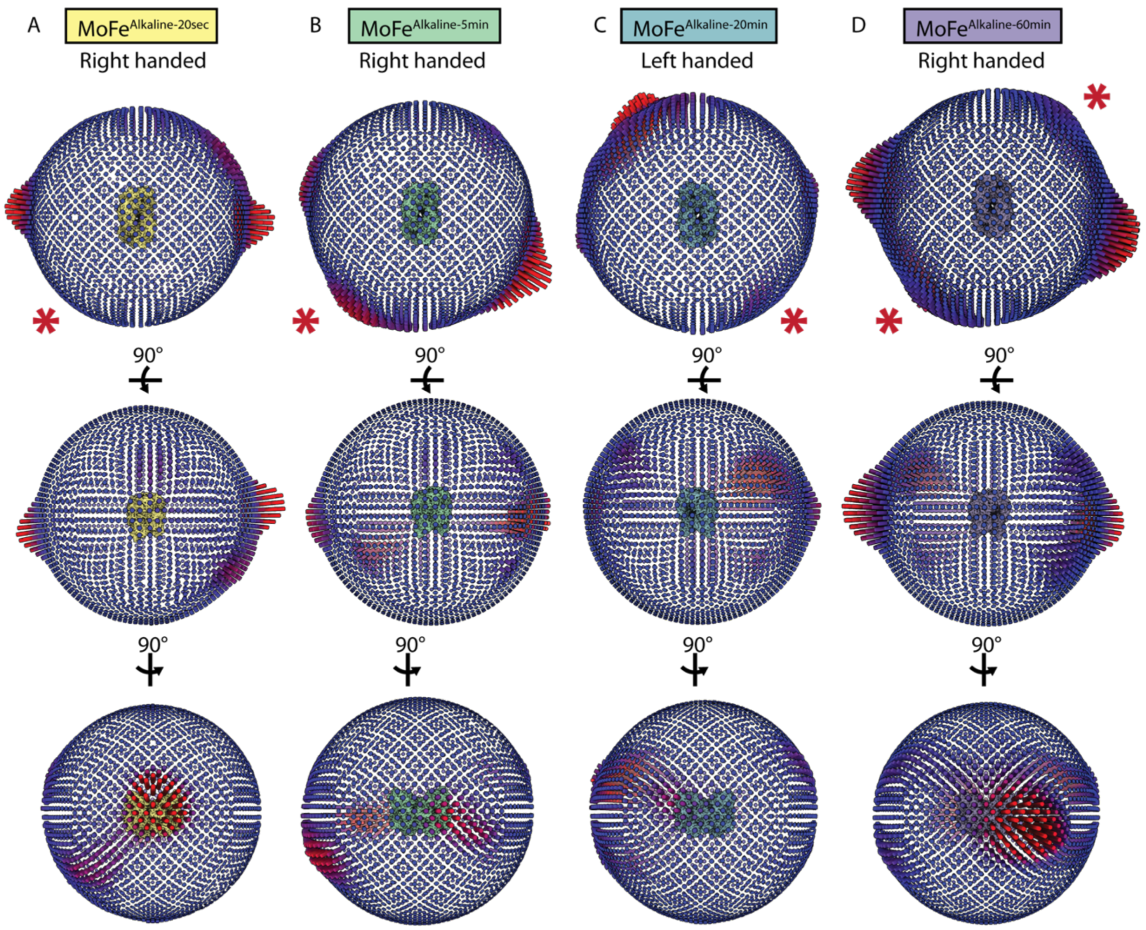

Supplementary Figure 10. Euler angle distribution maps for $MoFe^{Alkaline\text{-}20sec}$, $MoFe^{Alkaline\text{-}5min}$, $MoFe^{Alkaline\text{-}20min}$, and $MoFe^{Alkaline\text{-}60min}$. (A-D) Euler angle distribution maps are displayed as spheres about the central cryoEM density map, with the handedness of the initial map (right or left) indicated. Distributions of views at specific angles are indicated by histograms, with larger numbers of views indicated in red and higher bars. The locations of disordered active sites is indicated by red asterisks (*).

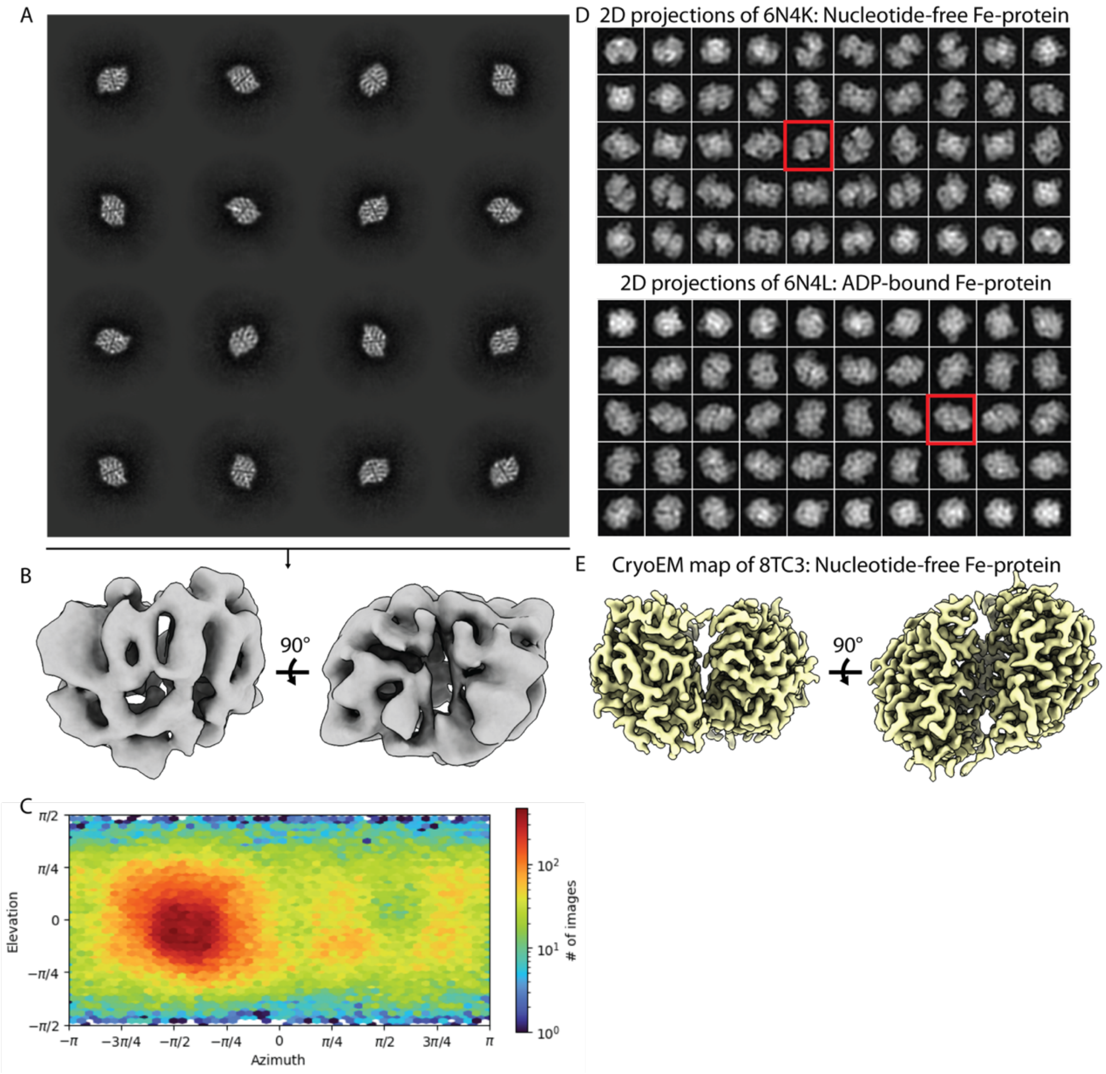

Supplementary Figure 11. Limited processing of nitrogenase Fe-protein particles in the MoFe$^{Alkaline\text{-}5min}$ time point. (A) 2D classes of nitrogenase Fe-protein particles. (B) Preliminary *ab initio* model of the Fe-protein. (C) Angular distribution heat map of the Fe-protein particles. (D) 2D projections of a 10 Å low-pass filtered volume of the nucleotide-free Fe-protein (PDB code 6N4K, top panel) and the ADP-bound Fe-protein (PDB code 6N4L, lower panel). Projections similar to the experiment views in panel A are boxed in red. (E) CryoEM density map of the nucleotide-free Fe-protein (PDB code 8TC3). The density of the Fe-protein more closely resembles the ADP bound state.

**Datasets**	**MoFe^{20sec}**	**MoFe^{5min}**	**MoFe^{20min}**	**MoFe^{60min}**	**MoFe$^{Alkaline\text{-}NafT}$**
PDB IDs	9CJE	9CJD	9CJC	9CJB	9CJF
Microscope	Titan Krios	Titan Krios	Titan Krios	Titan Krios	Titan Krios
Camera	Gatan K3 Summit	Gatan K3 Summit	Gatan K3 Summit	Gatan K3 Summit	Gatan K3 Summit
Magnification	130,000x	130,000x	130,000x	130,000x	130,000x
Voltage (kV)	300	300	300	300	300
Recording mode	counting	counting	counting	counting	counting
Frames/Movies	40	40	40	40	40
Total Electron dose (e-/Å2)	60	60	60	60	60
Defocus range (μm)	-0.8 to -3.0	-0.8 to -3.0	-0.8 to -3.0	-0.8 to -3.0	-0.8 to -3.0
Pixel size (Å)	0.65	0.65	0.65	0.65	0.65
Micrographs collected	10,734	10,583	11,082	11,034	5,349
Micrographs used					
Total extracted particles	7,061,454	7,057,624	5,947,375	9,249,178	558,639
Refined particles	113,613	411,562	335,402	787,718	78,918
Symmetry imposed	C1	C1	C1	C1	C1
Nominal Map Resolution (Å)	2.22	1.92	2.04	1.97	2.33
FSC threshold	0.143	0.143	0.143	0.143	0.143
masked/unmasked	2.2/2.2	1.9/1.9	2.0/2.0	1.7/1.8	2.2/2.3
Refinement					
Initial model used	8ENL	8ENL	8ENL	8ENL	8ENO
Number of atoms					
Protein	15,869	15,905	15,949	15,454	16,012
Ligand	ICS:2; CLF: 2; HCA:1; FE:2	ICS:2; CLF: 2; HCA:1; FE:2	ICS:2; CLF: 2; HCA:1; FE:2;	ICS:2; CLF: 2; FE:2	ICS:2; CLF: 2; FE:2; 1N7:2
MapCC (mask/box)	0.84/0.71	0.88/0.75	0.89/0.76	0.89/0.78	0.87/0.70
Map sharpening B-factor	47	48	54	54	51
R.m.s. deviations					
Bond lengths (Å)	0.003	0.003	0.004	0.004	0.002
Bond angles (º)	0.526	0.578	0.608	0.716	0.495
MolProbity score	1.56	1.31	1.35	1.35	1.40
Clashscore (all atom)	7.20	5.40	5.34	6.26	6.66
Rotamer outliers (%)	1.59	0.80	0.85	1.03	1.10
Ramachandran plot					
Favored (%)	97.94	97.92	97.72	98.66	98.14
Allowed (%)	2.01	2.08	2.28	1.34	1.86
Outliers (%)	0.05	0.00	0.00	0.00	0.00

Supplementary Table 1: Cryo-EM data collection, refinement, and validation statistics.
