# Supplementary material for: Structural evolution of nitrogenase states under alkaline turnover
Source: Nat Commun. 2024 Dec 2;15:10472. doi: 10.1038/s41467-024-54713-0 (PMC11612016; doi:10.1038/s41467-024-54713-0)
Supplement: Supplementary file 2 — Description of Additional Supplementary Files [file 41467_2024_54713_MOESM2_ESM.pdf]

Description of Additional Supplementary Files

File Name: Supplementary Movie 1
Description: Three-dimensional variability analysis (3DVA) of nitrogenase MoFe-protein under alkaline turnover. 3DVA was carried out on 2x downsampled particles following non-uniform refinement and symmetry expansion in cryoSPARC 4.4.1 with 3 modes over 20 iterations using a filter resolution of 7 Å. In these movies each of the modes is shown over the 20 frames.

File Name: Supplementary Movie 2
Description: Three-dimensional variability analysis (3DVA) of the $MoFe^{Alkaline\text{-}5min}$ suggests displacement of the FeMo-cofactor. $MoFe^{Alkaline\text{-}5min}$ 3DVA component 1 shown at 18 σ with the active site region beneath the αIII domain circled with a dashed line.
