# Supplementary material for: Structural evolution of nitrogenase states under alkaline turnover
Source: Nat Commun. 2024 Dec 2;15:10472. doi: 10.1038/s41467-024-54713-0 (PMC11612016; doi:10.1038/s41467-024-54713-0)
Supplement: Supplementary file 5 — Reporting Summary [file 41467_2024_54713_MOESM5_ESM.pdf]

nature portfolio

Corresponding author(s): Rebeccah A. Warmack
Douglas C. Rees

Last updated by author(s): Nov 12, 2024

Reporting Summary

Nature Portfolio wishes to improve the reproducibility of the work that we publish. This form provides structure for consistency and transparency in reporting. For further information on Nature Portfolio policies, see our Editorial Policies and the Editorial Policy Checklist.

Statistics

For all statistical analyses, confirm that the following items are present in the figure legend, table legend, main text, or Methods section.

n/a	Confirmed	
[ ]	[x]	The exact sample size (*n*) for each experimental group/condition, given as a discrete number and unit of measurement
[ ]	[x]	A statement on whether measurements were taken from distinct samples or whether the same sample was measured repeatedly
[x]	[ ]	The statistical test(s) used AND whether they are one- or two-sided<br>*Only common tests should be described solely by name; describe more complex techniques in the Methods section.*
[x]	[ ]	A description of all covariates tested
[x]	[ ]	A description of any assumptions or corrections, such as tests of normality and adjustment for multiple comparisons
[ ]	[x]	A full description of the statistical parameters including central tendency (e.g. means) or other basic estimates (e.g. regression coefficient) AND variation (e.g. standard deviation) or associated estimates of uncertainty (e.g. confidence intervals)
[x]	[ ]	For null hypothesis testing, the test statistic (e.g. *F*, *t*, *r*) with confidence intervals, effect sizes, degrees of freedom and *P* value noted<br>*Give P values as exact values whenever suitable.*
[x]	[ ]	For Bayesian analysis, information on the choice of priors and Markov chain Monte Carlo settings
[x]	[ ]	For hierarchical and complex designs, identification of the appropriate level for tests and full reporting of outcomes
[x]	[ ]	Estimates of effect sizes (e.g. Cohen's *d*, Pearson's *r*), indicating how they were calculated

Our web collection on statistics for biologists contains articles on many of the points above.

Software and code

Policy information about availability of computer code

Data collection	SerialEM version 4.1.1
Data analysis	GraphPad Prism version 10.0.0; Pymol 2.4.2; ChimeraX 1.7; cryoSPARC 4.4.1; Phenix 1.20.1-4487-000; RefMac5 in CCPEM 1.6.0; Coot 0.9.8.8

For manuscripts utilizing custom algorithms or software that are central to the research but not yet described in published literature, software must be made available to editors and reviewers. We strongly encourage code deposition in a community repository (e.g. GitHub). See the Nature Portfolio guidelines for submitting code & software for further information.

Data

Policy information about availability of data

All manuscripts must include a data availability statement. This statement should provide the following information, where applicable:

- Accession codes, unique identifiers, or web links for publicly available datasets
- A description of any restrictions on data availability
- For clinical datasets or third party data, please ensure that the statement adheres to our policy

The single particle cryoEM maps and models have been deposited into the PDB and EMDB for release upon publication. Datasets been deposited with the following PDB and EMDB codes: MoFeAlkaline-20sec (PDB ID 9CJE, https://doi.org/10.2210/pdb9CJE/pdb; EMD-45629, https://www.ebi.ac.uk/pdbe/entry/emdb/EMD-45629), MoFeAlkaline-5min (PDB ID 9CJD, https://doi.org/10.2210/pdb9CJD/pdb; EMD-45628, https://www.ebi.ac.uk/pdbe/entry/emdb/EMD-45628), MoFeAlkaline-20min (PDB ID 9CJC, https://doi.org/10.2210/pdb9CJC/pdb; EMD-45627, https://www.ebi.ac.uk/pdbe/entry/emdb/EMD-45627), MoFeAlkaline-60min (PDB ID 9CJB, https://doi.org/10.2210/pdb9CJB/pdb; EMD-45626, https://www.ebi.ac.uk/pdbe/entry/emdb/EMD-45626), and MoFeAlkaline-inactivated-NafT

complex (PDB ID 9CJF, https://doi.org/10.2210/pdb9CJF/pdb; EMD-45630, https://www.ebi.ac.uk/pdbe/entry/emdb/EMD-45630). CryoEM maps for 3DVA subclass maps MoFeAlkaline-5min-B ('cofactor out') and MoFeAlkaline-5min-D ('cofactor in') are included as supplementary maps with the MoFeAlkaline-5min deposition. All graphical data generated in this study are provided in the Source Data file.

Research involving human participants, their data, or biological material

Policy information about studies with human participants or human data. See also policy information about sex, gender (identity/presentation), and sexual orientation and race, ethnicity and racism.

Reporting on sex and gender	N/A
Reporting on race, ethnicity, or other socially relevant groupings	N/A
Population characteristics	N/A
Recruitment	N/A
Ethics oversight	N/A

Note that full information on the approval of the study protocol must also be provided in the manuscript.

Field-specific reporting

Please select the one below that is the best fit for your research. If you are not sure, read the appropriate sections before making your selection.

☒ Life sciences ☐ Behavioural & social sciences ☐ Ecological, evolutionary & environmental sciences

For a reference copy of the document with all sections, see nature.com/documents/nr-reporting-summary-flat.pdf

Life sciences study design

All studies must disclose on these points even when the disclosure is negative.

Sample size	Sample size of cryoEM datasets were determined by access to microscope time and a desire to achieve the highest resolution possible. Sample size of biochemical assays, including mPlum maturation experiments, were chosen on the basis of established laboratory practices and minimal requirements for statistical analysis.
Data exclusions	During cryoEM data processing, picked particles were excluded during data processing in cryoSPARC on the basis of standard criteria applied by this software.
Replication	Activity assays were performed in duplicate or triplicate under controlled conditions which allowed for assessment of reproducibility. Similar overall reconstructions from independent cryoEM datasets, support the reproducibility of the samples and the processing.
Randomization	For calculations of cryoEM map resolution, the cryoSPARC software randomly splits selected particles into two groups for gold standard Fourier Shell Correlation. Biochemical assays were not randomized as they do not include clinical trials or experiments with live organisms.
Blinding	Blinding is not relevant to our study. Data collection and quantification were performed with standardized procedures on dedicated equipment which excludes subjective bias.

Reporting for specific materials, systems and methods

We require information from authors about some types of materials, experimental systems and methods used in many studies. Here, indicate whether each material, system or method listed is relevant to your study. If you are not sure if a list item applies to your research, read the appropriate section before selecting a response.

Materials & experimental systems

n/a	Involved in the study	
☒	☐	Antibodies
☒	☐	Eukaryotic cell lines
☒	☐	Palaeontology and archaeology
☒	☐	Animals and other organisms
☒	☐	Clinical data
☒	☐	Dual use research of concern
☒	☐	Plants

Methods

n/a	Involved in the study	
☒	☐	ChIP-seq
☒	☐	Flow cytometry
☒	☐	MRI-based neuroimaging

Plants

Seed stocks	N/A
Novel plant genotypes	N/A
Authentication	N/A
